# Cannabidiol exerts antiepileptic effects by restoring hippocampal interneuron functions in a temporal lobe epilepsy model

**DOI:** 10.1111/bph.14202

**Published:** 2018-04-17

**Authors:** Archie A Khan, Tawfeeq Shekh‐Ahmad, Ayatakin Khalil, Matthew C Walker, Afia B Ali

**Affiliations:** ^1^ UCL School of Pharmacy London WC1N 1AX UK; ^2^ UCL Institute of Neurology London WC1N 3BG UK

## Abstract

**Background and Purpose:**

A non‐psychoactive phytocannabinoid, cannabidiol (CBD), shows promising results as an effective potential antiepileptic drug in some forms of refractory epilepsy. To elucidate the mechanisms by which CBD exerts its anti‐seizure effects, we investigated its effects at synaptic connections and on the intrinsic membrane properties of hippocampal CA1 pyramidal cells and two major inhibitory interneurons: fast spiking, parvalbumin (PV)‐expressing and adapting, cholecystokinin (CCK)‐expressing interneurons. We also investigated whether *in vivo* treatment with CBD altered the fate of CCK and PV interneurons using immunohistochemistry.

**Experimental Approach:**

Electrophysiological intracellular whole‐cell recordings combined with neuroanatomy were performed in acute brain slices of rat temporal lobe epilepsy in *in vivo* (induced by kainic acid) and *in vitro* (induced by Mg^2+^‐free solution) epileptic seizure models. For immunohistochemistry experiments, CBD was administered *in vivo* (100 mg·kg^−1^) at zero time and 90 min post status epilepticus, induced with kainic acid.

**Key Results:**

Bath application of CBD (10 μM) dampened excitability at unitary synapses between pyramidal cells but enhanced inhibitory synaptic potentials elicited by fast spiking and adapting interneurons at postsynaptic pyramidal cells. Furthermore, CBD restored impaired membrane excitability of PV, CCK and pyramidal cells in a cell type‐specific manner. These neuroprotective effects of CBD were corroborated by immunohistochemistry experiments that revealed a significant reduction in atrophy and death of PV‐ and CCK‐expressing interneurons after CBD treatment.

**Conclusions and Implications:**

Our data suggest that CBD restores excitability and morphological impairments in epileptic models to pre‐epilepsy control levels through multiple mechanisms to reinstate normal network function.

**Abbreviations**
ACSF: artificial CSF
AMPA: α‐amino‐3‐hydroxy‐5‐methyl‐4‐isoxazolepropionic acid
CB_1_: cannabinoid type‐1
CBD: cannabidiol
CCK: cholecystokinin
DG: dentate gyrus
FS: fast spiking
HW: width at half amplitude
KA: kainic acid
PFA: paraformaldehyde
PPR: paired pulse ratio
PV: parvalbumin
RT: rise time
SCA: Schaffer collateral‐associated
SE: status epilepticus
sEPSP: spontaneous EPSP
sIPSP: spontaneous IPSP
TBS‐T: Triton X‐100 in Tris‐buffered saline
TLE: temporal lobe epilepsy
TRPV: transient receptor potential vanilloid

## Introduction

Temporal lobe epilepsy (TLE) is the most common subtype of epilepsy in human patients (Wiebe, 2000) that results in stereotyped pathological changes including a common aetiology of hippocampal sclerosis. TLE can be examined in an array of animal TLE models, unlike other human epilepsies (Kandratavicius *et al.,* 2014; Levesque *et al.,* 2016). Factors such as cell death, neurogenesis and neuronal circuit reorganization have been attributed as causes of the disease process (Alexander *et al.,* 2016), as well as a prominent long‐standing hypothesis of the primary cause of epilepsy – ion channel abnormalities. Although ion channel defects can explain the onset of seizure events, they do not in all cases describe the physiological alterations that occur during the latent period of epileptogenesis – the process by which the brain develops epilepsy. The balancing of cellular events is performed by excitatory pyramidal cells and specialized inhibitory http://www.guidetopharmacology.org/GRAC/LigandDisplayForward?ligandId=1067 neurons that comprise approximately 10–15% of cortical neurons (Ascoli *et al.,* 2008), and it is generally accepted that the spread of epileptiform discharges involves a primary dysfunction of interneurons (Buhl *et al.,* 1996; Prince *et al.,* 1997; DeFelipe, 1999; Bausch, 2005). The fate of interneurons depends upon the subtype, and the diverse roles that interneurons play in epilepsy are becoming more evident; for example, seizure susceptibility is apparent in patients with reduced inhibitory circuitry (Wittner *et al.,* 2005; Liu *et al.,* 2014).

Several studies report the loss of hippocampal inhibitory interneurons in rodent models of epilepsy (Cavazos *et al.,* 1994; Fritschy *et al.,* 1999; Bouilleret *et al.,* 2000; Gorter *et al.,* 2001), as well as in human epileptic patients (Andrioli *et al.,* 2007). These observations are further corroborated by genetic studies using interneuron‐deficient transgenic mice suggesting that particular gene defects affect specific interneuronal development, resulting in seizures (Jones‐Davies *et al*., 2009). In general, these studies infer that the loss of interneurons has a negative impact on the balance of overall neuronal circuitry, which has been challenged by other research groups who suggest that specific interneuronal loss results in the preservation of lateral inhibitory circuits (Buckmaster and Jongen‐Relo, 1999). Therefore, it appears that the fate of interneurons in the epileptic brain, particularly the hippocampus, still remains largely ambiguous and unresolved.

Currently, there is a lack of effective treatments to prevent the progression of epileptogenesis, which is further complicated with the development of pharmacoresistance to conventional antiepileptic agents. Effective treatment of all types of epilepsy is therefore an unsolved problem in the field, resulting in a higher fraction of failed/inadequate seizure control in epileptic patients; thus, there is a continuing need for a better understanding of the cellular changes of epilepsy and epileptogenesis, as well as more effective and better tolerated antiepileptic drugs with better defined mechanisms of action.

One particular area of recent interest to researchers and clinicians has been the potential use of non‐psychoactive phytocannabinoids such as http://www.guidetopharmacology.org/GRAC/LigandDisplayForward?ligandId=4150) or its analogue cannabidivarin, also known as GWP42006 (Hill *et al.,* 2012a; Bialer *et al.,* 2015), in the treatment of epilepsy, particularly the more refractory, drug‐resistant forms (Ibeas Bih *et al.,* 2015; Devinsky *et al.,* 2017; Gaston and Friedman, 2017).

Previous studies have shown that CBD can dampen hippocampal epileptiform activity in both *in vitro* and *in vivo* animal models, without the psychotropic side effects associated with other phytocannabinoids (i.e. http://www.guidetopharmacology.org/GRAC/LigandDisplayForward?ligandId=2424, Δ^9^‐THC) that act through cannabinoid type‐1 (http://www.guidetopharmacology.org/GRAC/ObjectDisplayForward?objectId=56) receptors (Consroe and Wolkin, 1977; Consroe *et al.,* 1982; Kogan and Mechoulam, 2007; Jones *et al.,* 2010; 2012), making the cannabinoid system an interesting and novel therapeutic target for epilepsy. These observations have been corroborated by recent human clinical studies, where CBD reduced the convulsive seizure frequency by 50%, giving hope for patient groups with uncontrollable epilepsy where other drug treatments have failed, namely, in a complex childhood epilepsy disorder associated with high mortality rate, Dravet syndrome (Devinsky *et al.,* 2017). This syndrome is associated with sodium channel mutations that may have specific effects on interneurons, reducing interneuronal excitability.

Like most antiepileptic drugs, CBD is thought to have multiple targets, and further investigations are clearly required to elucidate the cellular mechanisms through which CBD exerts its anti‐seizure effects as well as any possible neuroprotectant activity. Studying single channels does not inform us about whether a drug would decrease the excitation of interneurons or pyramidal cells; for example, we know that antiepileptic drugs such as sodium channel blockers affect both excitation and inhibition that can worsen epilepsy when there is a deficit in interneuronal excitability as in Dravet's syndrome (Rubinstein *et al*., 2015). Therefore, we have taken an alternative, novel approach and addressed the functional impact of CBD treatment on excitatory and inhibitory local circuits in epilepsy. Our general hypothesis is that the intrinsic membrane properties of pyramidal cells and the two major subclasses of interneurons: fast spiking (FS), parvalbumin (PV)‐expressing and adapting, cholecystokinin (CCK)‐expressing interneurons are altered, leading to impaired local circuit activity in the hippocampus during epilepsy, and that the activity of these cells and their local circuit function will be restored by CBD, due to a neuroprotective effect. Using electrophysiological techniques, we initially investigated whether CBD could alter the epilepsy‐induced synaptic and biophysical properties of pyramidal cells, PV‐ and CCK‐expressing interneurons, in an established rodent model of epilepsy. We then investigated whether CBD treatment could alter the fate of these two major interneuron types in the epileptic brain using anatomical studies.

## Methods

### Animals

Animal studies are reported in compliance with the ARRIVE guidelines (Kilkenny *et al*., 2010; McGrath and Lilley, 2015). All experiments were performed in accordance with British Home Office regulations under the Animal Scientific Procedure Act 1986. Home Office project licences held by the PI (AA: PPL No. 7007558) and were approved by internal and external ethical review panels. Adult male Sprague–Dawley rats, between postnatal days 42 and 56 and weighing between 200 and 250 g, were used in this study. All animals were housed on a 12 h light‐dark cycle, with food and water available *ad libitum*.

### Epileptic models

Two seizure models were used, *in vivo* kainic acid (KA)‐induced epilepsy and an *in vitro* Mg^2+^‐free hippocampal brain slice model (see below). For the KA model, animals were rendered epileptic by the administration of a single i.p. injection of KA, at a dose of 10 mg·kg^−1^ (Ben‐Ari and Cossart, 2000). The severity of seizures was scored using the Racine (1972) scale, and seizures of grade 5 (i.e. rearing, bilateral forelimb clonus and falling with loss of postural control) were accepted. The onset of spontaneous seizures occurred between 30 and 100 min after the injection of KA. The animals were further monitored until the full development of status epilepticus (SE). To minimize mortality related to SE, sustained seizures were terminated by a single injection of http://www.guidetopharmacology.org/GRAC/LigandDisplayForward?ligandId=3364 at a dose of 10 mg kg‐1, i.p., as required by the Home Office regulations. The duration of SE was measured based on behavioural manifestations, and onset of SE was considered to occur when the rat experienced full motor seizures with loss of postural control and falling. Rats were maintained for a further 2 weeks post‐KA injection to ensure the development of spontaneous seizures, before using them either for electrophysiological *in vitro* experiments or for neuroanatomical studies.

For neuroanatomical studies, rats were randomly assigned to one of the four experimental groups: (i) healthy control (*n* = 5 rats); (ii) epileptic_vehicle [single injection of vehicle (10% DMSO/saline), at a time interval of 2 weeks post‐SE onset, *n* = 6 rats]; (iii) CBD_time 0 [single injection of 100 mg·kg^−1^ CBD (Tocris Bioscience, UK), zero time interval of the SE onset, *n* = 6 rats]; and (iv) CBD_time 90 (single injection of 100 mg·kg^−1^ CBD, 90 min interval post‐SE onset, *n* = 6). Substances were injected i.p. The dose of CBD injected into rats was approximately the ED_50_ for animal seizure models in the National Institute of Neurological Disorders and Stroke screening (Jones *et al.,* 2010).

All rats were anaesthetized *via* gas inhalation of http://www.guidetopharmacology.org/GRAC/LigandDisplayForward?ligandId=2401 (Fluothane) followed by sodium phenobarbital injection (60 mg·kg^−1^, i.p.) for electrophysiological and neuroanatomical studies. The level of anaesthesia was monitored using pedal, tail pinch reflexes, rate, depth and pattern of respiration through observation and colour of mucous membrane and skin.

### Electrophysiology for *in vitro* recordings

Rats were anaesthetized and perfused with a sucrose‐containing artificial CSF (ACSF) solution that consisted the following (in mM): 248 sucrose, 3.3 KCl, 1.4 NaH_2_PO_4_, 2.5 CaCl_2_, 1.2 MgCl_2_, 15 glucose and 25.5 NaHCO_3_, bubbled with 95% O_2_ and 5% CO_2_. Following decapitation and removal of the brain, coronal slices of cortex, 300 μm thick, were cut in ice‐cold ACSF using an automated vibratome (Leica, Germany). This standard ACSF contained (in mM) the following: 121 NaCl, 2.5 KCl, 1.3 NaH_2_PO_4_, 2 CaCl_2_, 1 MgCl_2_, 20 glucose and 26 NaHCO_3_, equilibrated with 95% O_2_ and 5% CO_2_. Brain slices were placed in a submerged chamber and super‐perfused with ACSF at a rate of 1–2 mL·min^−1^ for 1 h at room temperature (20–23°C) prior to recording. To generate the *in vitro* Mg^2+^‐free epileptic model, brain slices were incubated in oxygenated (95% O_2_–5% CO_2_) ACSF, containing (in mM) 121 NaCl, 2.5 KCl, 1.25 NaH_2_PO_4_, 2 CaCl_2_, 26 NaHCO_3_ and 20 glucose, for approximately 2 h.

Paired whole‐cell somatic recordings were obtained between CA1 pyramidal cells (for excitatory connections) and between interneurons and postsynaptic pyramidal cells (for inhibitory connections). Patch electrodes with resistances of 8–11 MΩ were made from filamented borosilicate glass capillaries (Harvard Apparatus, UK) and filled with a solution containing (in mM): 134 K gluconate, 10 HEPES, 10 phosphocreatine, 2 Na_2_ATP, 0.2 Na_2_GTP and 0.2% w ^.^ v^‐1^ biocytin. Neurons were selected for recording based on the shape of their soma using video microscopy under near infrared differential interference contrast illumination and further characterised by their electrophysiological properties obtained from a series of 500 ms depolarising and hyperpolarising current pulses. Action potential parameters were measured from responses of depolarising current steps (+50–150 pA, 500 ms), which induced a single or trains of action potentials. The input resistance and membrane time constant were determined from voltage changes in response to hyperpolarising current steps (−100 pA, 500 ms).

Unitary EPSPs or IPSPs were elicited by a depolarising current step into the presynaptic neuron (+0.05 nA, 5–10 ms) repeated at 0.33 Hz. Spontaneous PSPs were recorded from cells routinely held at −55 mV membrane potential by injection of steady depolarising current. Recordings were carried out in the current clamp mode of operation (SEC 05LX npi amplifiers; npi electronics, Germany), low pass filtered at 2 KHz and digitised at 5 KHz using a CED 1401 interface (Cambridge Electronic Design, UK). Input resistance was monitored throughout experiments by means of a hyperpolarising current step (−0.001 nA, 10 ms). Signal (Cambridge Electronic Design) was used to acquire recordings and generate current steps. The peak EPSP/IPSP amplitudes, 10–90% rise time (RT) and width at half amplitude (HW) measurements were obtained from averages including 100–200 unitary synaptic events. Apparent failures of transmission were assigned a value of 0 mV and were not included in averages.

For *in vitro* pharmacology studies, drugs such as CBD (5, 8 and 10 μM) and a selective antagonist of CB_1_ receptors AM4113 (1 μM; Tocris Bioscience) were bath‐applied.

### Immunohistochemistry

After anaesthesia, intracardial perfusion was performed, with PBS, followed by 4% paraformaldehyde (PFA) solution in 0.1 M PBS, pH 7.4 (200 mL^.^100 g^‐1^ of body weight). The animals were then decapitated, and the brains were rapidly removed and fixed in a solution containing of 4% w ^.^v ^‐1^ PFA, 0.025% v ^.^ v^‐1^ glutaraldehyde, 0.2% v ^.^ v ^‐1^ picric acid in 0.1 M PBS, overnight. Coronal brain sections of 100 μm thick were cut using a vibratome (Vibroslice, Camden Instrument, Loughborough, UK) and washed in 0.1% Triton X‐100 in TBS (TBS‐T), followed by incubation in 1% hydrogen peroxide aqueous solution for 30 min. After further rinses in TBS‐T, sections were incubated in PBS containing 10% normal goat serum (Sigma‐Aldrich, USA) for 1 h at room temperature. This followed incubation in the primary antibody solution, containing 1:5000 diluted anti‐PV antibody raised in rabbit (SWANT, UK) or 1:10 000 diluted anti‐CCK antibody raised in mouse (gift from Gordon Ohing, Cure, UCLA) and 1% horse serum in TBS‐T, for 24 h at 4°C. After washes in TBS‐T, the sections were then incubated in secondary biotinylated anti‐rabbit or anti‐mouse antibodies raised in goat (Vector Laboratories, UK), diluted at 1:500 for 24 h at 4°C. This followed washes in TBS‐T and a further incubation in avidin‐biotin‐horseradish peroxidase complex (Vector Laboratories) solution, for 2 h at room temperature. The sections were then washed further in TBS‐T and processed for 3‐3‐diaminobenzidine and subsequently dehydrated and mounted (see Ali, 2007).

### Reconstruction of immunolabelled neurons and analysis

Anatomical maps of immunopositive somata of PV and CCK neurons in the hippocampal formation were manually drawn from three consecutive 100 μm‐thick sections using a drawing tube attached to a Leica DMR microscope under ×10 magnification; the images were scanned and digitized to superimpose the 300 μm sections to perform cell density analysis using software, Image J (version 1.49, RBS, Maryland, USA). PV‐ and CCK‐expressing interneurons were reconstructed using a drawing tube connected to a Leica DMR microscope with ×40 objective to investigate anatomical changes and for Sholl analysis. Only complete, fully impregnated interneurons with no apparent truncation of the dendritic arbor were included in this study. Sholl analysis was performed on the reconstructed images using a preformed template in Image J with a radius step size of 10 μm. The area under the Sholl curve can be used as a measure of dendritic complexity, and changes in this value are indicative of morphological alterations (Ristanovic *et al.,* 2006).

### Statistical analyses

The data and statistical analysis comply with the recommendations on experimental design and analysis in pharmacology (Curtis *et al*., 2015). All data values are given as the mean ± SD, unless otherwise stated. Student's two‐tailed, paired *t*‐test was used to compare two groups of data. For comparisons between more than two groups of data, a one‐way ANOVA statistical test was used, followed by a *post hoc* Tukey's test. All statistical analyses were conducted using the statistical package Origin Pro 2016 SR1. Statistical significance was accepted where *P* < 0.05. The ‘*n*’ values represent the number of observations and the number of animals used, unless otherwise stated.

### Nomenclature of targets and ligands

Key protein targets and ligands in this article are hyperlinked to corresponding entries in http://www.guidetopharma-cology.org, the common portal for data from the IUPHAR/BPS Guide to PHARMACOLOGY (Harding *et al.,* 2018), and are permanently archived in the Concise Guide to PHARMACOLOGY 2017/18 (Alexander *et al.,* 2017a,b,c).

## Results

This study initially focused on investigating the effects of CBD on the synaptic and biophysical properties of neurons followed by neuroanatomical investigation in the CA1 hippocampal subfield.

### CBD alters unitary synaptic connections, favouring reduced seizure activity

To investigate whether CBD alters unitary synaptic events, we first investigated unitary excitatory connections between two pyramidal cells and then the inhibition elicited by presynaptic FS, PV and presynaptic adapting CCK cells onto postsynaptic pyramidal cells in the CA1 region of the hippocampus. There are various subclasses of adapting CCK cells in CA1; we focused on the Schaffer collateral‐associated (SCA) interneurons which are abundant in CA1 (Ali, 2007). All interneuron subtypes were confirmed anatomically.

#### CBD reduces excitation at pyramidal cells

**Figure 1:**
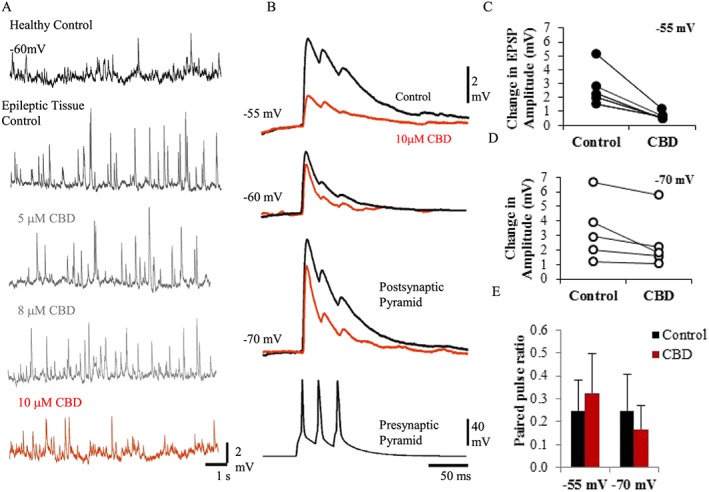
CBD dampens excitation. (A) Spontaneous EPSPs, recorded at a membrane potential of −60 mV in healthy control tissue and KA model of epileptic tissue. Concentrations of 5, 8 and 10 μM CBD were bath‐applied. sEPSP frequency and peak amplitudes were only significantly changed with 10 μM of CBD. (B) Unitary recording between two pyramidal cells synaptically connected. Triple presynaptic action potentials elicited by the pyramidal cell resulted in EPSPs that displayed synaptic depression recorded at three different membrane potentials (−70, −60 and −55 mV). These EPSPs were reduced in amplitude and duration following bath application of 10 μΜ CBD (red traces). (C, D) Plots of change in peak average amplitude of EPSPs per synaptic connection (*n* = 5) with bath application of CBD recorded at −55 and −70 mV. CBD had a more pronounced action at more positive membrane potentials. (E) Plot of PPR (second‐amplitude EPSP/first‐amplitude EPSP) at −55 and −70 mV; no significant change in PPR was measured in CBD‐treated cells.

Initial experiments were performed to assess the effects of CBD on spontaneous EPSPs (sEPSPs; Figure 1A). The mean frequency and amplitude of sEPSPs recorded at a membrane potential of −60 mV in healthy control and KA‐induced epileptic tissue was 0.52 ± 0.23 Hz (0.57 ± 0.83 mV in amplitude, *n* = 5 pyramidal cells, *n* = 3 animals) and 1.74 ± 0.54 Hz (1.62 ± 0.96 mV in amplitude, *n* = 5 pyramidal cells, *n* = 3 animals) respectively. Only significant reductions in the sEPSP frequency and amplitude were observed with 10 μM bath application of CBD, which restored the sEPSPs properties to control healthy levels of 0.55 ± 0.42 Hz (0.86 ± 0.71 mV, *P* < 0.05, *n* = 5; Figure 1A).

For unitary connections, the probability of finding two CA1 pyramidal cells that were synaptically connected was low. Only one in 12 pairs of pyramidal cells tested yielded a synaptic connection. We found that CBD exerted its action at unitary excitatory synapses by reducing the amplitude of EPSPs (Figure 1B). EPSPs elicited in excitatory pyramidal cells by other pyramidal cells were reduced (*n* = 5). The EPSP amplitude and duration were both decreased at all postsynaptic membrane potentials. However, the effect of CBD was more pronounced at a depolarized membrane potential (−55 mV), reducing the amplitude significantly from 2.75 ± 1.40 mV (control) to 0.62 ± 0.31 mV after 10 μM CBD bath application (*n* = 5, *P <* 0.05). In comparison, at a more negative postsynaptic membrane potential of −70 mV, the mean change of the amplitudes was reduced from 3.33 ± 2.10 to 2.48 ± 1.90 mV with bath application of CBD (*n* = 5, *P >* 0.05). This suggests that the EPSPs elicited at postsynaptic pyramidal cells are significantly affected by CBD at more depolarized levels of excitation. Synaptic connections typically possessed a non‐conventional voltage relationship in the current study (Figure 1B–D), where EPSP amplitudes decreased in peak amplitude from a holding potential of −70 to −60 mV; however, beyond a membrane holding a potential of −58 mV, the EPSP increased in amplitude and duration. These connections also typically displayed synaptic depression, as indicated by the average paired pulse ratio (PPR) (second‐amplitude EPSP/first‐EPSP amplitude) at −55 mV, 0.25 ± 0.13 (*n* = 5), which was not significantly changed with bath application of CBD, 0.32 ± 0.17 (Figure 1E), suggesting a postsynaptic mechanism of action. CBD did not significantly change the EPSP RT or the duration at either membrane potentials, which were in the range of 2.5 and 3.5 ms and 14 and 21 ms respectively.

#### Enhanced inhibition

**Figure 2:**
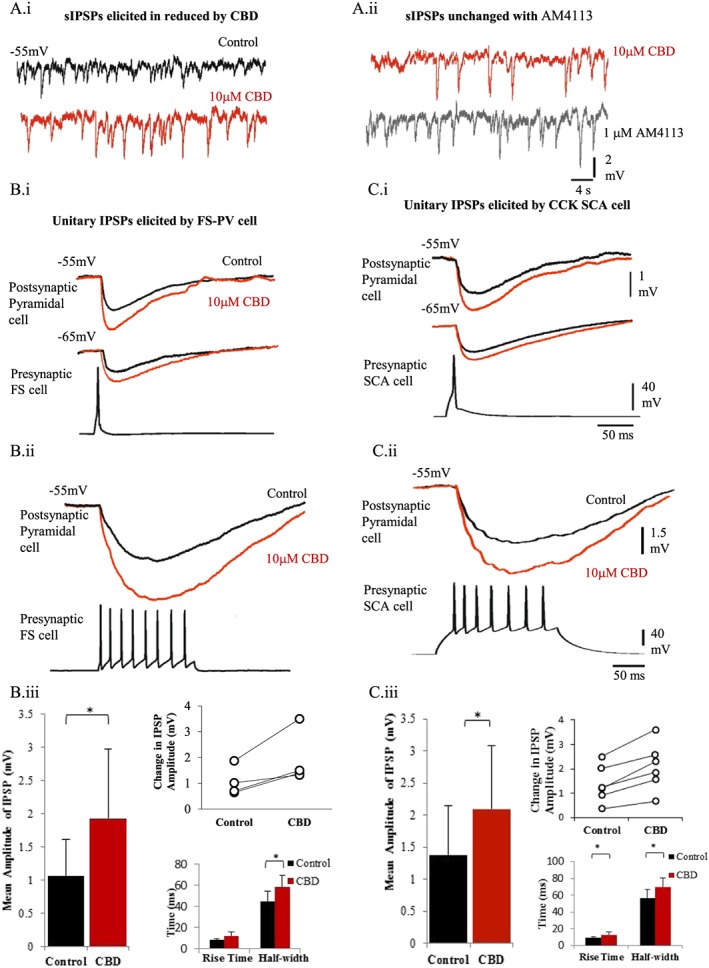
CBD enhances inhibition. (A, panels i, ii) sIPSPs, recorded at a membrane potential of −55 mV. The frequency and peak amplitudes of sIPSPs increased with CBD (10 μM) and remained unchanged with subsequent addition of a selective CB_1_ receptor antagonist AM4113 (1 μM). (B, panels i, ii) Unitary connection between a presynaptic FS PV containing interneuron (FS‐PV) and a postsynaptic excitatory cell is shown. Averaged IPSPs are shown as black traces (in control) and after bath application 10 μM CBD (20–30 min, red traces). (B, panels i, ii.) IPSPs elicited by CCK, SCA interneuron onto postsynaptic pyramidal cells. The top traces in (A) and (B) left panel show the averaged IPSPs elicited by one presynaptic action potential, whereas the bottom panel shows the inhibition elicited by a train of presynaptic interneuron action potentials; as a result, there is summation of the inhibitory event. (C, panels i,– ii) Plots show the average change in IPSP amplitudes, IPSP RTs and width at half amplitude elicited by FS‐PV and CCK, SCA presynaptic interneurons onto pyramidal cells with bath application of CBD. The line plots show change in peak average IPSP amplitude with CBD per individual synaptic connection.

In contrast to excitation, spontaneous IPSPs (sIPSP) recorded in pyramidal cells and unitary IPSPs elicited by FS and adapting CCK (SCA cells) cells onto postsynaptic pyramidal cells held at −55 mV were both enhanced by bath application of CBD (Figure 2A). The mean sIPSPs amplitude and frequency were enhanced by 45 ± 7.8 and 38.5 ± 10%, of the control values with bath application of 10 μM CBD respectively (*n* = 4 postsynaptic pyramidal cells, *n* = 3 animals). These events were unchanged with subsequent bath application of a selective antagonist of CB_1_ receptors, AM41131 (1 μM), shown in Figure 2A.

Unitary IPSPs elicited by FS cells displayed a fast RT (Figure 2B, panels i–iii; 10–90% RTs range, 7–10 ms; HW range; 30–51 ms), with an average peak amplitude of 1.06 ± 0.56 mV (*n* = 4). This amplitude increased to 1.93 ± 1.04 mV and thus by 182% of control amplitude with bath application of CBD (*n* = 4).

The IPSPs elicited by adapting SCA cells displayed slower RTs (10–90% RTs range, 8–11 ms; HW range; 40–65 ms; see Figure 2C, panels i–iii). The average peak amplitude was 1.37 ± 0.76 mV (*n* = 6), which was also enhanced to 2.09 ± 0.98 mV of control amplitude by CBD, a significant increase by 150 ± 25% of control (*n* = 6, *P* < 0.05).

### CBD exerts a cell type‐specific alteration of membrane properties of CA1 neurons

The intrinsic membrane properties of neurons reveal information about how a cell will relay information in the network. It is well documented that principal pyramidal cells show hyperactivity in epileptic tissue; hence, we investigated the intrinsic membrane properties of FS (PV), adapting (CCK) and pyramidal cells in healthy rats and in two different seizure models: the *in vivo* KA model of epilepsy and the *in vitro* Mg^2+^‐free brain slice model (see Table 1 for quantitative data and statistical significance). In the KA model of epilepsy, all three cell types were significantly affected in their membrane properties, indicative of the severity of this model in recapitulating human TLE.

**Table 1:** Electrophysiological properties of healthy control, Mg^2+^‐free and KA‐induced epileptic hippocampal cells under CBD treatment

Healthy control rats						
	Pyramidal cells (*n* = 13)		Adapting cells (*n* = 11)		FS cells (*n* = 9)	
Subclass of cells	Control	CBD	Control	CBD	Control	CBD
AP Amp (mV)	76.02 ± 2.24	73.60 ± 1.77	81.45 ± 2.51	84.62 ± 6.23	71.66 ± 1.23	70.45 ± 2.02
AP HW (ms)	1.83 ± 0.07	3.35 ± 1.56	1.43 ± 0.14	1.52 ± 0.15	1.28 ± 0.16	1.28 ± 0.16
AP threshold (mV)	26.11 ± 1.76	27.59 ± 2.98	20.96 ± 2.04	23.84 ± 2.72	26.15 ± 3.43	18.72 ± 1.37*
AP AHP Amp (mV)	4.94 ± 0.80	5.47 ± 0.87	6.40 ± 0.59	6.50 ± 0.74	11.50 ± 0.82	12.02 ± 0.47
Input resistance (MΩ)	290.08 ± 15.75	241.21 ± 14.10*	305.42 ± 35.11	245.21 ± 29.62*	289.60 ± 38.00	396.84 ± 52.64*
TC (ms)	13.58 ± 1.90	10.42 ± 0.36*	12.82 ± 1.24	11.47 ± 0.82*	10.06 ± 0.44	14.23 ± 0.49*
No. of spikes at +150 pA	8.90 ± 0.61	5.40 ± 0.35*	12.09 ± 1.06	6.45 ± 0.76*	15.50 ± 2.88	22.25 ± 2.96^**^

Mg^2+^‐free epileptic model						
	Pyramidal cells (*n* = 5)		Adapting cells (*n* = 5)		FS cells (*n* = 4)	
Subclass of cells	Control	CBD	Control	CBD	Control	CBD
AP Amp (mV)	78.36 ± 3.41	76.93 ± 3.83	88.26 ± 6.42	71.75 ± 18.71	75.54 ± 9.83	72.33 ± 8.27
AP HW (ms)	2.01 ± 0.18	2.20 ± 0.38	1.22 ± 0.16	1.32 ± 0.14	1.58 ± 0.43	1.52 ± 0.41
AP threshold (mV)	24.80 ± 1.83	25.80 ± 2.99	25.85 ± 1.54	21.27 ± 5.58	28.70 ± 5.80	21.24 ± 2.55
AP AHP Amp (mV)	6.05 ± 0.74	5.37 ± 0.48	6.54 ± 0.83	6.05 ± 1.74	2.64 ± 1.29	4.49 ± 1.95
Input resistance (MΩ)	308 ± 16.55	227.60 ± 21.35*	289.06 ± 36.67	244.92 ± 37.83*	351.45 ± 46.36	396.20 ± 65.74
TC (ms)	14.06 ± 1.23	10.03 ± 0.68*	8.28 ± 1.73	8.74 ± 0.72	8.75 ± 0.60	12.08 ± 0.63
No. of spikes at +150 pA	12.80 ± 1.20	4.25 ± 0.22*	10.20 ± 0.66	4.60 ± 0.51*	10.75 ± 1.49	17.75 ± 2.87

KA‐induced epileptic model						
	Pyramidal cells (*n* = 12)		Adapting cells (*n* = 12)		FS cells (*n* = 7)	
Subclass of cells	Control	CBD	Control	CBD	Control	CBD
AP Amp (mV)	79.42 ± 2.81	78.33 ± 2.55	81.78 ± 3.34	77.67 ± 1.66	74.61 ± 2.39	75.46 ± 2.41
AP HW (ms)	2.14 ± 0.10	2.43 ± 0.18	1.59 ± 0.13	1.84 ± 0.11	1.34 ± 0.21	1.32 ± 0.18
AP threshold (mV)	27.61 ± 2.65	27.31 ± 3.57	26.76 ± 2.87	27.41 ± 2.75	19.12 ± 1.67	15.74 ± 1.85*
AP AHP Amp (mV)	2.42 ± 0.25	2.46 ± 0.33	7.23 ± 1.70	4.62 ± 0.64	12.00 ± 1.09	10.59 ± 1.57
Input resistance (MΩ)	313.84 ± 25.44	272.04 ± 25.42*	364.49 ± 43.35	285.65 ± 43.12*	372.63 ± 43.55	411.34 ± 51.50
TC (ms)	14.98 ± 0.74	10.58 ± 1.16*	10.25 ± 0.56	7.99 ± 0.42*	9.50 ± 0.81	12.21 ± 0.77*
No. of spikes at +150 pA	14.18 ± 0.28	4.92 ± 0.48*	12.36 ± 1.60	7.64 ± 1.23*	10.00 ± 0.71	21.43 ± 3.21*

AP, action potential; Amp, amplitude; HW, half‐width; AHP, after‐hyperpolarization; TC, time constant; FS, fast spiking.

Results are expressed as mean ± SEM.

* *P* < 0.05.

Student's two‐tailed paired *t*‐test was used to compare between control and CBD treatment conditions of different biophysical properties of each cell‐type.

**Figure 3:**
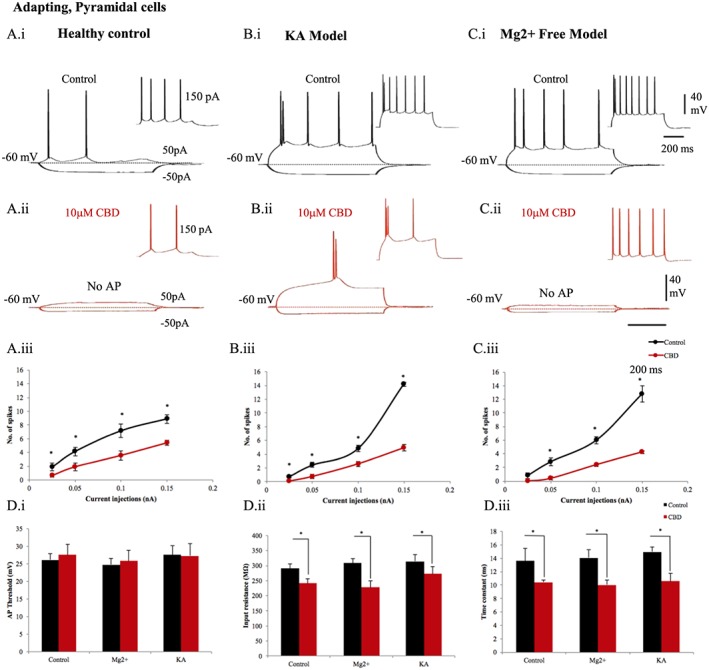
Pyramidal cell's hyperactive membrane properties are restored by CBD. The intrinsic membrane properties of pyramidal cells recorded in healthy rats in (A, panel i) control and (A, panel ii) after 10 μΜ CBD bath application. Similarly, pyramidal cells recorded brain slices in (B, panel i), (B, panel ii) Mg^2+^‐free and from (C, panel i), (C, panel ii) KA epileptic models. The input–output curves displayed a pseudo‐linear relationship between the number of spikes generated by pyramidal cells of (A, panel iii) healthy rodents (*n* = 13), (B.iii) Mg^2+^‐free (*n* = 5) and (C, panel iii) KA (*n* = 12) epileptic models with increasing current injections. The firing of pyramidal cells was decreased after application of CBD. (D) Bar plots representing intrinsic membrane properties of pyramidal cells in Mg^2+^‐free (*n* = 5) and KA epileptic models (*n* = 12) in comparison to healthy rodents (*n* = 13). (D, panel i) CBD produced no apparent changes of action potential (AP) threshold of pyramidal cells. (D, panel ii) The input resistance and (D, panel iii) time constant were decreased by CBD. Results are expressed as mean ± SEM. *P ≤ 0.05.

Principal pyramidal cells display adapting action potential properties with slow kinetics compared to the other hippocampal neuronal subpopulations studied in healthy control rats (Table 1, Figure 3). After 20 min of bath application of 10 μM CBD, significant changes in the intrinsic membrane properties included a reduced number of spikes and a decreased input resistance in healthy control rats (Figure 3). In general, hippocampal pyramidal cells were hyper‐excited in both the *in vivo* KA model and the *in vitro* Mg^2+^‐free model, as evidenced by a higher number of action potential discharge and enhanced input resistance recorded at a fixed membrane potential of −60 mV (Figure 3D). In these models, bath application of CBD also resulted in significant changes by reducing the number of spikes, the input resistance and time constant of the cells. A rightward shift of the control output curves representing number of evoked spikes plotted against increasing depolarizing current injections (Figure 3A, panel iii, B, panel iii, C, panel iii) indicated the suppression of excitability induced by CBD in pyramidal cells. CBD caused a negligible increase in mean action potential threshold in pyramidal cells; also, the mean action potential half‐width, amplitude and spike after‐hyperpolarization (AHP) amplitude showed no statistically significant differences in the experimental groups. Interestingly, bath application of CBD also resulted in 10 mV hyperpolarization of the membrane potential.

### Effect of CBD on the intrinsic membrane properties of adapting cells that express neuropeptide CCK

**Figure 4:**
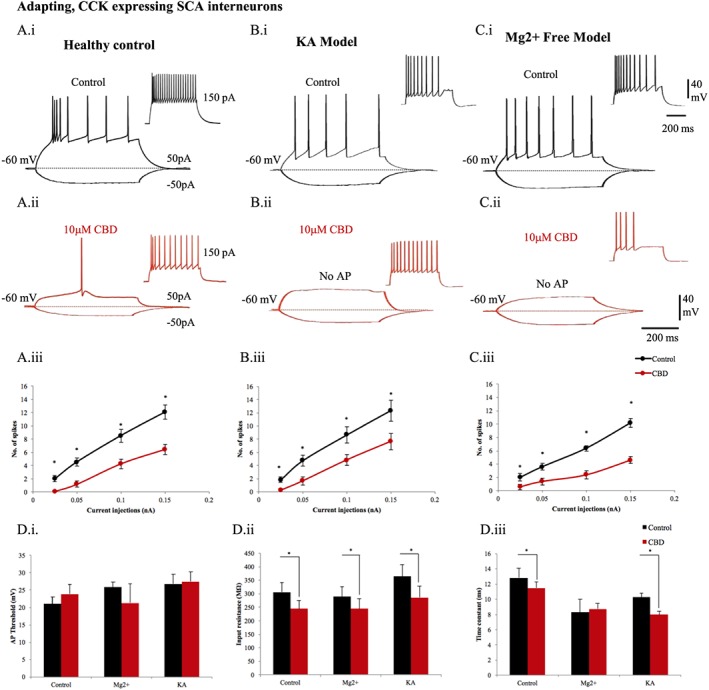
CBD reduces intrinsic excitability of CCK adapting interneurons in CA1. The membrane properties of SCA interneurons, which were CCK positive, were recorded in healthy rats (A, panel i) in control and (A, panel ii) after 10 μΜ CBD bath application. Similar recordings are shown in Mg^2+^‐free condition (B, panel i), (B, panel ii) and in the KA epileptic model (C, panel i), (C, panel ii). The input–output curves displayed a pseudo‐linear relationship between the number of spikes generated by adapting cells of (A, panel iii) healthy rodents (*n* = 11), (B, panel iii) Mg^2+^‐free (*n* = 5) and (C, panel iii) KA (*n* = 12) epileptic models with increasing current injections. The number of action potentials of adapting interneurons was decreased after application of CBD. (D) Bar plots representing intrinsic membrane properties of adapting cells in Mg^2+^‐free (*n* = 5) and KA epileptic models (*n* = 12) in comparison to healthy rodents (*n* = 11). (D, panel i) CBD produced no apparent changes of action potential (AP) threshold of adapting cells. (D, panel ii) The input resistance and (D, panel iii) time constant were decreased by CBD in the disease models. Results are expressed as mean ± SEM. *P ≤ 0.05.

In general, CCK SCA cells displayed intermediate action potential duration (0.3–0.5 ms in spike width; Figure 4). In healthy control rats, CBD (10 μΜ) altered the membrane properties of adapting, CCK‐expressing cells, by significantly decreasing the mean number of spikes generated in response to depolarizing current injections of 500 ms (Figure 4A, panel i, to C, panel ii; see also Ali, 2007). A decreased mean input resistance and time constant was observed in healthy control SCA cells with CBD treatment (Figure 4D). These changes were also consistent in the Mg^2+^‐free and KA seizure models (see Table 1). A rightward shift illustrated in the control scatter plot (representing number of spikes at increasing positive current injections) was observed indicating the reduction of the CCK SCA cell excitability with CBD treatment in healthy controls and both epileptic/seizure models (Figure 4A, panel iii, B, panel iii, C, panel iii). No significant differences of mean action potential amplitude, half‐width, threshold or AHP were observed after bath application of CBD in any of the experimental groups. Bath application of CBD also resulted in a 10 mV hyperpolarization of the membrane potential as seen in pyramidal cells.

**Figure 5:**
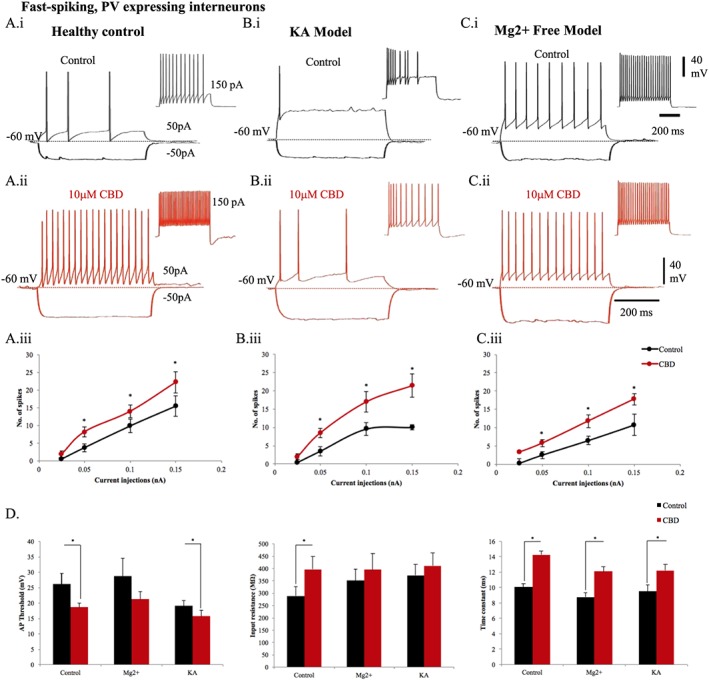
CBD enhances excitability of FS‐PV interneurons in the CA1 region of the hippocampus. The membrane properties of FS‐PV interneurons recorded in healthy rats (A, panel i) in control and (A, panel ii) after a 10 μΜ CBD bath application. Similarly, FS‐Pv cells were also recorded in Mg^2+^‐free (B, panel i), (B, panel ii) and in KA epileptic models (C, panel i), (C, panel ii). The input–output curves displayed a pseudo‐linear relationship between the number of spikes generated by FS PV cells of (A, panel iii) healthy rodents (*n* = 9), (B, panel iii) Mg^2+^‐free (*n* = 4) and (C, panel iii) KA (*n* = 7) epileptic models with increasing current injections. The firing of FS‐PV interneurons was dramatically increased after application of CBD. (D) Bar plots representing intrinsic membrane properties of FS PV cells in Mg^2+^‐free (*n* = 4) and KA epileptic models (*n* = 7) in comparison to healthy rodents (*n* = 9). (D, panel i) CBD produced a reduction of action potential (AP) threshold of FS PV cells. (D, panel ii) The input resistance and (D, panel iii) time constant were increased by CBD in healthy rodents and the seizure models. Results are expressed as mean ± SEM. *P ≤ 0.05.

FS interneurons, typically expressing PV, showed characteristic fast action potential kinetics and high frequency of action potential discharge (with little spike frequency adaption and accommodation), consistent with a fast membrane time constant (Table 1, Figure 5). The general health of FS interneurons recorded was substantially impaired when recorded particularly in the KA model, which showed a reduced action potential threshold, increased spike frequency adaption and accommodation, with respect to control FS cells, with the same amount of step depolarization (Figure 5A, panel i, to C, panel ii). However, these cells were unable to sustain a high frequency of firing (Figure 5B, panel i), suggesting impaired membrane properties. Following a 20 min bath application of CBD (10 μM), the number of action potentials increased with the same amount of depolarizing current injections as control conditions observed in brain slices of healthy control rats and in the Mg^2+^‐free and KA epilepsy models (Figure 5). This observation was consistent with significant increases in input resistance for the same hyperpolarizing current steps injected as in control conditions and an increased time constant (Figure 5D). Higher input resistance shows a greater change in membrane voltage in the presence of CBD, suggesting a favourable enhanced excitability of FS PV cells with this drug. Enhanced firing of FS cells with bath application of CBD and input resistance was also correlated with a significant reduction of mean action potential threshold and faster mean time constants (Figure 5D), which was consistent in all three experimental conditions studied (see Table 1). Similar to pyramidal cells and adapting SCA cells, bath application of CBD also resulted in a 10 mV hyperpolarization of the membrane potential in FS cells.

### Changes in PV‐ and CCK‐expressing interneuron density and morphology in epilepsy and after CBD treatment

**Figure 6:**
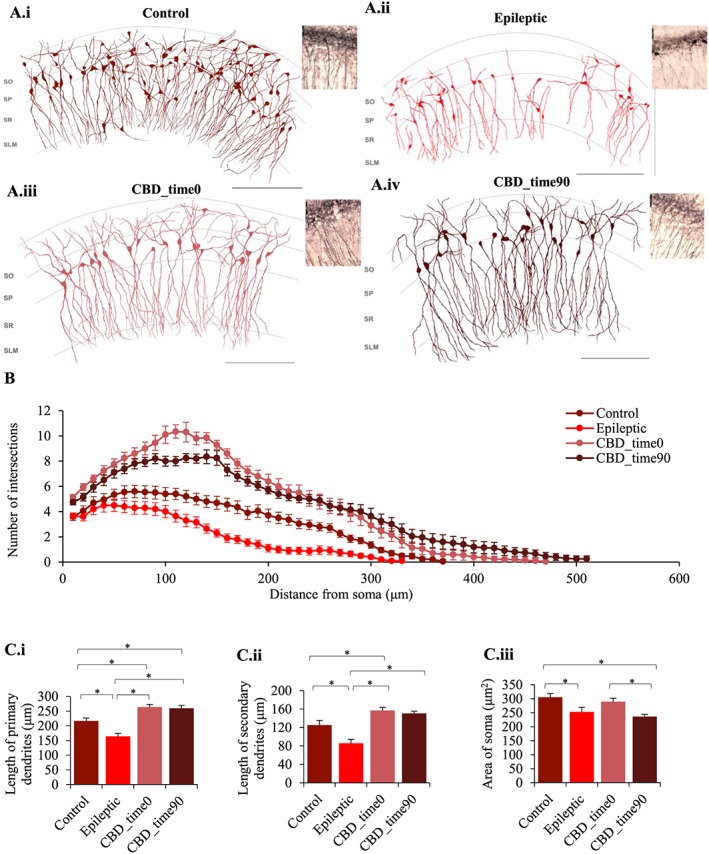
CBD halts PV cell death in the hippocampi of KA‐induced epileptic rats. (A) Reconstructions and photomicrographs of all the PV neurons present in the CA1 hippocampal subfield of a randomly picked rat in (A, panel i) control, (A, panel ii) epileptic, (A, panel iii) CBD‐treated at zero time (CBD_time0) and (A, panel iv) at 90 min (CBD_time90) post‐SE conditions using a 40× magnification in a light microscope with an attached drawing tube, from three consecutive 100‐μm‐thick coronal sections of rat hippocampi. The CA1 hippocampal subfield including, SO, SP, and stratum radiatum (SR) and stratum lacunosum moleculare (SLM). All photomicrographs taken of PV neurons were from brains sections processed using immunoperoxidase labelling. Scale bars set at 250 μm for all the reconstructions and 50 μm for all the photomicrographs. (B, panel i) Sholl plot exhibiting the pattern of dendritic arborisation of PV neurons in the CA1 hippocampal subfield of rats in all experimental conditions, control, epileptic and CBD‐treated at zero time (CBD_time0) and at time 90 min (CBD_time90) post‐SE (*n* = 20, for all). Morphometric analyses of PV neurons *(n* = 20) were performed, which are represented as bar graphs comparing the (C, panel i) length of primary dendrites (μm), (C, panel ii) secondary dendrites (μm) and (C, panel iii) area of soma (μm^2^) between all the experimental groups. Results are expressed as mean ± SEM (*P ≤ 0.05; one‐way ANOVA with *post hoc* Tukey's test).

**Figure 7:**
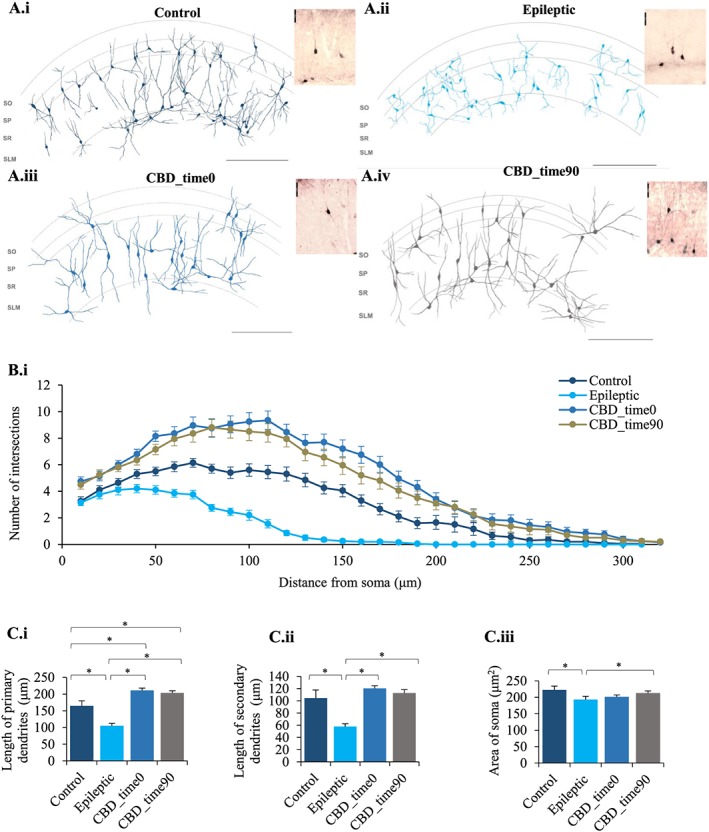
CBD prevents CCK cell death in the hippocampi of KA‐induced epileptic rats. (A) Reconstructions and photomicrographs of all the CCK neurons present in the CA1 hippocampal subfield of a randomly picked rat in (A, panel i) control, (A, panel ii) epileptic, (A, panel iii) CBD‐treated at zero time (CBD_time0) and (A.iv) at 90 min (CBD_time90) post‐SE conditions using a 40× magnification in a light microscope with an attached drawing tube, from three consecutive 100‐μm‐thick coronal sections of rat hippocampi. All photomicrographs taken of CCK neurons were from brains sections processed using immunoperoxidase labelling. Scale bars set at 250 μm for all the reconstructions and 50 μm for all the photomicrographs. (B, panel i) Sholl plot exhibiting the pattern of dendritic arborization of CCK neurons in the CA1 hippocampal subfield of rats in all experimental conditions, control, epileptic and CBD‐treated at zero time (CBD_time0) and at time 90 min (CBD_time90) post‐SE (*n* = 20, for all). Morphometric analysis of CCK neurons (*n* = 20) were performed which are represented as bar graphs comparing the (C, panel i) length of primary dendrites (μm), (C, panel ii) secondary dendrites (μm) and (C, panel iii) area of soma (μm^2^) between all the experimental groups. Results are expressed as mean ± SEM (*P ≤ 0.05; one‐way ANOVA with *post hoc* Tukey's test).

The mean cell density of the PV‐ and CCK‐expressing neurons in three subfields of the rat hippocampal formation including CA1, CA3 and the dentate gyrus (DG) was first studied and compared between control animals and the KA epileptic model. In all three subfields, there was a significant reduction in the density of PV‐ and CCK‐expressing neurons in epileptic rats in comparison to control rats (see Table 2). CA1 was examined in more detail, and there was a decrease in the number of PV‐ and CCK‐expressing cells in stratum oriens (SO), stratum pyramidale (SP), stratum radiatum and stratum lacunosum moleculare of epileptic rats. This reduction in the cell densities was reduced by CBD at both treatment time points (CBD_time0 and CBD_time90). Figures 6 and 7 illustrate the dendritic morphology of PV‐ and CCK‐expressing interneurons in control rats and KA‐induced epileptic rats before and after treatment with CBD. Morphometric analyses of these reconstructed neurons were performed to compare the changes between control and epileptic rats after CBD treatment at time points, 0 and 90 min of SE. Sholl analysis, a measure of the distribution of dendritic complexity as a function of distance from soma (Sholl, 1953), revealed a marked compromise of soma size and dendritic arborization for both PV‐ and CCK‐expressing cells between control and the epileptic group (Table 2), which was also prevented by CBD treatment, irrespective of the treatment time points.

**Table 2:** Morphometric and cell count analyses of hippocampal PV‐ and CCK‐expressing cells in epilepsy and after CBD‐treatment of KA‐induced epileptic rats

	Cell density in hippocampal subfields mm^‐3^			Normalized cell density in hippocampal layers mm^‐3^				Length of dendrites (μm)		Area of soma (μm^2^)
	CA1	CA3	DG	SO	SP	SR	SLM	Primary	Secondary	
Control (*n* = 20)	569 ± 37	539 ± 120	644 ± 104	589 ± 139	2355 ± 225	216 ± 34	169 ± 16	216 ± 10	125 ± 10	305 ± 13
Epileptic (*n* = 20)	172 ± 18	170 ± 15	269 ± 44	128 ± 38	847 ± 116	49 ± 8	73 ± 18	163 ± 10	86 ± 8	253 ± 16
CBD_time0 (*n* = 20)	368 ± 41	329 ± 36	702 ± 53	511 ± 99	1899 ± 257	101 ± 20	89 ± 17	264 ± 9	157 ± 7	290 ± 12
CBD_time90 (*n* = 20)	357 ± 43	318 ± 15	618 ± 39	543 ± 119	2036 ± 246	111 ± 11	36 ± 16	259 ± 10	151 ± 5	236 ± 8
Control‐epileptic	*	*	*	*	*	*	*	*	*	*
Control‐CBD_time0	*	–	–	–	–	*	*	*	*	–
Control‐CBD_time90	*	–	–	–	–	*	*	*	–	*
Epileptic‐CBD_time0	*	–	*	–	*	–	–	*	*	–
Epileptic‐CBD_time90	*	–	*	*	*	–	–	*	*	–
CBD_time0‐CBD_time90	–	–	–	–	–	–	–	–	–	*

CCK‐expressing cells										
	Cell density in hippocampal subfields mm^‐3^			Normalized cell density in hippocampal layers mm^‐3^				Length of dendrites (μm)		Area of soma (μm^2^)
	CA1	CA3	DG	SO	SP	SR	SLM	Primary	Secondary	
Control (*n* = 20)	305 ± 42	272 ± 46	484 ± 121	171 ± 34	563 ± 132	316 ± 62	308 ± 109	164 ± 15	104 ± 14	222 ± 12
Epileptic (*n* = 20)	89 ± 14	107 ± 7	140 ± 8	40 ± 8	130 ± 30	147 ± 34	42 ± 10	105 ± 7	58 ± 5	193 ± 10
CBD_time0 (*n* = 20)	283 ± 21	344 ± 38	375 ± 40	231 ± 55	585 ± 85	143 ± 14	317 ± 40	211 ± 7	120 ± 4	201 ± 6
CBD_time90 (*n* = 20)	307 ± 32	311 ± 33	323 ± 38	239 ± 56	565 ± 96	147 ± 20	394 ± 84	203 ± 7	113 ± 6	213 ± 6
Control‐epileptic	*	*	*	–	–	*	–	*	*	*
Control‐CBD_time0	–	–	–	–	–	*	–	*	–	–
Control‐CBD_time90	–	–	–	–	–	*	–	*	–	–
Epileptic‐CBD_time0	*	*	*	*	*	–	*	*	*	–
Epileptic‐CBD_time90	*	*	–	*	*	–	*	*	*	*
CBD_time0‐CBD_time90	–	–	–	–	–	–	–	–	–	–

Results are expressed as mean ± SEM.

* *P* < 0.05.

A one‐way ANOVA with *post hoc* Tukey's test was used for statistical analyses. Statistical significance was tested between control, epileptic and CBD‐treated groups.

In PV‐expressing cell densities, significant recovery of PV was apparent in SO and SP in the epileptic rats, and 87 and 92% of control PV‐expressing cells in SO were recovered at CBD_time0 and CBD_time90 respectively. Similarly, a recovery of 80 and 86% of control PV‐expressing cells was seen in SP at CBD_time0 and CBD_time90, respectively, suggesting a better recovery after 90 min of SE.

PV‐expressing cells present in epileptic rats exhibited a Sholl profile of a downward shift of the curve from the control curve (Figure 6A). In contrast, the Sholl profile of PV‐expressing neurons of CBD‐treated groups shifted upwards, with a slight skewness of the curve to the left, from the control and epileptic groups (Table 2; Figure 6B). Furthermore, analyses of mean length and number of primary and secondary dendrites of PV cells revealed a significant reduction in the epileptic animals in comparison to control. This dendritic truncation seemed halted and elongated beyond control levels when epileptic rats were treated with CBD (Figure 6C, panels i, ii).

CCK‐expressing cell densities also significantly recovered in all the three subfields of the hippocampus after CBD treatment, which was on par with healthy control levels with no significant differences between the control and CBD‐treated groups (Table 2, Figure 7).

The expression of CCK‐expressing cells in epileptic rats displayed a similar trend in the change of dendritic morphology during epilepsy and after treatment with CBD (Figure 7). The Sholl profile of the CCK‐expressing cells of epileptic rats revealed a downward shift, suggesting a deterioration in the dendritic arborisation in comparison to the control rats. This shift was reversed by CBD treatment, at both time 0 and time 90, resulting in the Sholl profile curves shifting upwards, with a slight skewness towards the left, beyond the control levels. The dendrites of CBD‐treated CCK cells in comparison, regardless of the time of treatment administration, appeared to ramify mainly further from the soma with maximum branching at a distance of approximately 114% increase in length (Figure 7C).

## Discussion

In this report, we investigated the cellular mechanisms of action of CBD, which affected synaptic transmission, and intrinsic excitability of pyramidal cells as well as PV‐expressing interneurons and CCK‐expressing interneurons. The pattern of progression of epilepsy in the KA model was reminiscent of the development of TLE in humans, particularly after a precipitating insult, such as focal brain trauma or febrile seizures, which are some of the most common causes of secondary TLE (Cavalheiro *et al.,* 1982; Ben‐Ari, 1985; Ben‐Ari and Cossart, 2000). Therefore, this model is of particular relevance to the human condition and offers a very suitable platform to study the mechanisms of the disease and also to evaluate potential novel treatments such as cannabinoids.

Furthermore, our data suggest a neuroprotective role for CBD, since treatment with CBD rescued the morphological pathology of PV‐ and CCK‐expressing interneurons induced by epilepsy.

### CBD reduces hyper‐excitation via modulating excitation and inhibition

CBD consistently reduced synaptic transmission at excitatory synapses between pyramidal neurons. Interestingly, the reduction in EPSPs was dependent on the membrane potential and the amount of presynaptic excitation. For example, excitatory cells firing at low frequencies at a less excitable state (−70 mV) were not significantly affected by CBD but had the most significant affect at high firing rates held at a more excitable membrane potential (−55 mV), which may be secondary to the voltage‐dependent properties of CBD receptor targets or to a voltage‐dependent block of those targets by CBD. As GPCR 55 (http://www.guidetopharmacology.org/GRAC/FamilyDisplayForward?familyId=114)‐mediated Ca^2+^ elevation in hippocampal glutamatergic terminals has been shown to increase the probability of transmitter release, the antagonizing action of CBD could also contribute to the reduced glutamate release and, as a consequence, reduce the aberrant glutamate‐mediated excitation in the hippocampus (Sylantyev *et al.,* 2013). However, as indicated by the unchanged PPR of EPSPs after CBD treatment in the present study, we suggest a postsynaptic mechanism of action for this cannabinoid.

GPR55 activation also leads to inhibition of http://www.guidetopharmacology.org/GRAC/ObjectDisplayForward?objectId=564) channels, thereby increasing neuronal excitability (Lauckner *et al.,* 2008). Perhaps by activating http://www.guidetopharmacology.org/GRAC/ObjectDisplayForward?objectId=561), CBD reduces spontaneous and unitary EPSPs in a voltage‐dependent manner, which would help dampen seizure‐related hyperexcitability in the hippocampus and thus limit the damage associated with excitotoxicity. This could also contribute to the membrane hyperpolarisation seen in pyramidal cells and the two classes of interneurons studied in the presence of CBD. One other possibility is that CBD has a similar effect to THC in activating postsynaptic G‐protein coupled inwardly rectifying potassium channels, http://www.guidetopharmacology.org/GRAC/FamilyIntroductionForward?familyId=74 **.** CBD has been reported to activate transient receptor potential vanilloid (http://www.guidetopharmacology.org/GRAC/FamilyDisplayForward?familyId=78) channel subtypes, which are ligand‐gated, non‐selective cation (Na^+^, Mg^2+^ and Ca^2+^) channels (Billeter *et al.,* 2014), at various potencies. Agonism of human http://www.guidetopharmacology.org/GRAC/ObjectDisplayForward?objectId=507 expressed in HEK293 cells by CBD has been popularly reported with different potencies (3–10 μm) (Bisogno *et al.,* 2001; De Petrocellis *et al.,* 2011). The existing literature surrounding the role of TRPV1 in epilepsy is conflicted between different studies which indicate that activation of TRPV1 has no involvement (von Ruden *et al.,* 2015), a proconvulsant (Manna and Umathe, 2012) or an anticonvulsant (Chen *et al.,* 2013; Gonzalez‐Reyes *et al.,* 2013) effect in epilepsy. Whether CBD is directly modulating http://www.guidetopharmacology.org/GRAC/FamilyDisplayForward?familyId=75 or targeting a novel postsynaptic cannabinoid receptor or alternatively working in synchrony with other previously established targets, for example, GRP55 or TRPV receptors (De Petrocellis *et al.,* 2011; 2012; Hill *et al.,* 2012b; Iannotti *et al.,* 2014), remains to be investigated.

### CBD enhances inhibition on postsynaptic pyramidal cells

The present study reports for the first time that CBD enhances inhibition elicited by fast spiking PV and CCK interneurons that display spike frequency accommodation and adaptation, referred to as ‘adapting CCK cells’ connected to postsynaptic pyramidal cells. This will ultimately lead to a reduction in pyramidal cell activation and reduce hyper‐excitability. At present, we do not know the mechanism of action of this effect at inhibitory synapses. However, previous evidence suggests that CBD does not exert its pharmacological effects *via* the GPCRs of the endocannabinoid system, CB_1_ and CB_2_, to the same extent as THC, as it may possess a low affinity with little to almost no agonist activity on these receptors (Pertwee, 2008). However, we found no change in the CBD‐mediated enhancement of inhibition with the selective CB_1_ receptor antagonist AM4113; therefore, the involvement of CB_1_ receptors in our *in vitro* experiments can be eliminated.

### CBD changes the innate firing properties of specific neurons in the hippocampus

Interneurons have a characteristic innate firing pattern, which tells us how fast information between neurons is relayed that may be a contributing factor in epileptogenesis and ictogenesis. This study provides evidence, for the first time, to suggest that CBD alters the firing of neurons in a cell type‐specific fashion. In both the KA and Mg^2+^‐free seizure models of epilepsy, FS cells exhibited a more excitable state in their membrane properties with CBD, evidenced by a lower threshold of firing action potentials, an increase in membrane time constant and input resistance, which will ultimately raise the neuron's voltage level quicker and in turn result in the cell being more readily available to fire action potentials. The precise mechanism of the increased excitability of FS cells by CBD is unknown; it could be hypothesized that CBD interacts with the membrane leak channels altering the membrane permeability to sodium ions, or perhaps CBD directly modulates the calcium buffering capacity of PV within FS cells changing the rate of firing. The FS cells typically innervate the soma and dendrites of pyramidal cells (Kawaguchi *et al.,* 1987; Freund and Buzsaki, 1996) and therefore are able to directly inhibit excessive excitatory discharge of pyramidal cells, enabling them to efficiently entrain and synchronize neuronal networks. The effect of CBD resulting in an enhanced neuronal discharge of FS cells will be a favoured outcome in epilespy as a direct increase of inhibitory effects which may contribute towards the supression of the disease progression. In contrast, CBD favoured a reduced firing of adapting, CCK interneurons that play a role in network modulation, as well as pyramidal cell firing, evidenced by a decrease in membrane time constant and input resistance in both the KA and Mg^2+^‐free models. Incidentally, a significant loss of principal pyramidal neurons in both human and rodent epileptic hippocampi has been reported (Nadler *et al.,* 1978; Babb *et al.,* 1989), despite others reporting additional axonal sprouting of pyramidal cells, which will lead to enhancement of excitatory networks between pyramidal neurons, and probably explains the hyperactivity (Perez *et al.,* 1996; Esclapez *et al.,* 1999). Whether the CBD effects in reducing pyramidal cell hyperactivity reported here lead to a perseveration of pyramidal cell structure needs further investigation.

Evidence suggests that a small conductance Ca^2+^‐activated K^+^ channel underlies the generation of the slow spike AHP, which is important for spike frequency accommodation and thus the firing output of the neurons (Lancaster and Nicoll, 1987; Sah and Isaacson, 1995; Sah and Faber, 2002); although the exact channel associated with this effect still remains unknown, some studies suggest that an inhibition of http://www.guidetopharmacology.org/GRAC/ObjectDisplayForward?objectId=560) and K_v_7.2 (*KCNQ2*) channels blocks the slow AHP of hippocampal pyramidal neurons (Shah *et al.,* 2006). Whether CBD activates these channels directly, enhancing the slow AHP effect to dampen cell excitability, is yet to be fully investigated.

### CBD halts morphological pathology of two major interneuron subclasses in epilepsy

Our overall findings show a significant reduction in the density in both PV‐ and CCK‐expressing cells in the hippocampus proper, including the CA1, CA3 and the DG. Furthermore, we observed morphological changes in these interneurons, which included distorted, shrunken somata and altered, shorter dendritic arborisation and ramification, and an overall reduction in dendritic complexity. These distortions in the morphology and overall reduction in the cell densities were halted by treatment of rats with CBD.

The reduction in cell densities are consistent with previous studies in epileptic rodents (Best *et al.,* 1993; Buckmaster and Dudek, 1997; Dudek *et al.,* 2002; Wyeth *et al.,* 2010; Jiang *et al.,* 2016); however, others have shown a loss of hippocampal PV‐expressing cells in tissue obtained from sclerotic patients despite the increase of total neuronal density in the subiculum (Andrioli *et al.,* 2007). Whether the loss of PV‐ and CCK‐immunoreactivity is due to cell death or the lack of expression of the neuromarkers is still under debate. For example, despite the loss of PV‐expressing cell bodies and dendrites, a preservation of the axon terminals together with inhibitory input of pyramidal cells was reported in human TLE using electron microscopy (Wittner *et al*., 2001).

The physiological relevance of the morphological changes in these neurons would impact on the functional integrity of these neurons including disturbances in connectivity and how information is processed (Weinberger, 1999). As a result, compromised fine‐tuning of interneuron function in the epileptic hippocampus may lead to the reduced inhibition of pyramidal cells (Lancaster and Wheal, 1984; Cornish and Wheal, 1989; Kobayashi and Buckmaster, 2003). Together, the potentiation of excitatory mechanisms and the ineffective control of persistent excitation would synergistically facilitate the generation and propagation of epileptiform activity (de Lanerolle *et al.,* 1989).

The mechanisms of how CBD exerts a neuroprotective action could be related to reduced oxidative stress. In the present study, administration of KA to rats to induce epilepsy caused persistent activation of glutamate AMPA/kainate receptors triggering a sustained neuronal influx of Na^+^ and Ca^2+^, causing prolonged depolarization and prolonged Ca^2+^ influx into the cytoplasm. However, previous studies suggest that CBD exerts an activity‐dependent biphasic control over Ca^2+^ levels (Ryan *et al.,* 2009); hence, it is likely that in our experiments, CBD treatment of epileptic rats exerted a neuroprotective function by preventing intracellular Ca^2+^ overloading during hyperactivity, thus halting oxidative stress. This would trigger mitochondrial oxidation (Hajnoczky *et al.,* 2006), contributing to the degenerative process of neurons (Liang *et al.,* 2000; Patel, 2004; Waldbaum *et al.,* 2010).

We observed an increase in PV‐ and CCK‐expressing cell densities, as well as an increase in the dendritic length after CBD treatment, and while the notion of adult neurogenesis has been controversial, it is evidenced by studies that show modulation of various receptors and their signal transduction pathways, such as GPCRs (Kaplan and Hinds, 1977; Doze and Perez, 2012; Goncalves *et al.,* 2016). For example, neurogenesis in both embryonic and cultured adult hippocampal neural stem and progenitor cells has been shown *via* the activation of GPCRs, CB_1_ receptors and ERK signalling by CBD and other synthetic cannabinoids (Jiang *et al.,* 2005). Furthermore, CBD has been implicated in CB_1_ receptor‐mediated proliferation and maturation of progenitor cells (Wolf *et al*., 2010). While the established CBD actions *via* CB_1_ receptors are few, it could be postulated that the proneurogenic effects of CBD in the reported results are a consequence of sequential activation GPCRs and CB_1_ receptors. Therefore, CBD could be potentiating a morphological self‐repair mechanism of the surviving PV‐ and CCK‐expressing neurons *via* neurogenesis in the adult epileptic hippocampus of rats.

In conclusion, the impaired morphology of interneurons and pyramidal cells reported here and by others following seizure activity probably contributes to impaired inhibitory mechanisms in epileptic hippocampi, and existing evidence of reorganization of the neuronal circuitry probably contributes to the generation and propagation of hyper‐synchronous activity of large neuronal populations that underlie epileptiform activity. A decrease in firing properties of pyramidal cells in the hippocampus caused by CBD suggests that it reduces excitability, while an increase in firing properties at specific interneurons suggests that CBD will enhance inhibition at some synapses, ultimately leading to a rebalance of excitation, thus supporting our hypothesis. The precise cellular mechanism of the pre‐ and postsynaptic receptors involved in these changes, however, requires further investigation. Pharmacoresistance to conventional anti‐epileptic agents is an unresolved problem in the field of epilepsy resulting in a higher fraction of failed treatment in epileptic patients. The mechanisms of action by which currently available pharmacological anti‐epileptic drugs work, for example, blocking voltage‐gated sodium channels, only suppress neuronal discharge by a rate‐dependent mechanism. Na channel blockers, such as carbamazepine and valproate, are widely used in the treatment of TLE, but the dangerous side effects and the associated increased risk of suicide often limit the use of the drug or result in the use of a second anti‐epileptic treatment (adjuvant therapy). CBD is a multi‐targeted naturally‐derived plant compound with little to no side effect profile. This makes CBD a desirable candidate for the treatment of TLE that could play a vital antiepileptic and neuroprotective role in the epileptic CNS by enhancing selective inhibition and dampening excitation, which would ultimately reduce seizure activity and prevent neuronal death.

## Author contributions

A.A.K. performed single whole‐cell electrophysiological experiments, neuroanatomical experiments and data analysis and contributed in preparing the manuscript. T.S.‐A. performed *in vivo* experiments to generate the KA epilepsy model. A.K. performed *in vivo* experiments to generate the KA epilepsy model. M.C.W. provided funding to generate *in vivo* experiments to generate the KA epilepsy model and assisted in preparing the manuscript. A.B.A. designed and coordinated the project, performed all paired whole‐cell recordings, single whole‐cell recordings and data analysis and prepared the manuscript.

## Conflict of interest

The authors declare no conflicts of interest.

## Declaration of transparency and scientific rigour

This http://onlinelibrary.wiley.com/doi/10.1111/bph.13405/abstract acknowledges that this paper adheres to the principles for transparent reporting and scientific rigour of preclinical research recommended by funding agencies, publishers and other organisations engaged with supporting research.
